# Electrochemical Reduction of CO_2_ to C2 Hydrocarbons Using Cu 3D Nanostructures

**DOI:** 10.3390/ma18174210

**Published:** 2025-09-08

**Authors:** Birutė Serapinienė, Evaldas Naujalis, Algirdas Selskis, Jurga Juodkazytė, Rimantas Ramanauskas

**Affiliations:** Center for Physical Sciences and Technology (FTMC), Saulėtekio 3, LT-10257 Vilnius, Lithuania; birute.serapiniene@ftmc.lt (B.S.); evaldas.naujalis@ftmc.lt (E.N.); selskisa@gmail.com (A.S.); jurga.juodkazyte@ftmc.lt (J.J.)

**Keywords:** CO_2_ electrochemical reduction, Cu 3D electrode, structure-properties relationship, facet distribution, grain boundaries

## Abstract

Although Cu 3D structures are widely used in electrocatalytic practice, this electrode has not been studied enough in relation to the electrochemical transformation of CO_2_ to C2 products. Cu foam samples were deposited from acidic solutions with varying concentrations of primary components (H_2_SO_4_, CuSO_4_, and Cl^−^ ions) with the aim of determining the relationship between catalyst structure and activity/selectivity in producing C2 gaseous compounds during CO_2_ electrochemical reduction. The deposited samples were characterized using SEM and electrochemical techniques, including Pb underpotential deposition (UPD), to determine the contribution of crystal facets. The most efficient electrodes were found to be those deposited in a solution without Cl^−^ additives. Their effectiveness was related to the shape and size of the crystallites forming the branches. These crystallites create a spatial structure that supports C-C coupling and C2 gaseous compound formation. The higher catalytic activity and selectivity of this electrode may also be related to its lower Cu(111) facet input to the overall facet distribution and its higher number of structural defects. Despite the higher electrochemically active surface area of samples deposited in the presence of Cl^−^ ions, their lower activity is related to structural characteristics that cause possible mass transfer limitations.

## 1. Introduction

The main challenges facing high performance in electrochemical reduction of CO_2_ (CO2ER) on Cu electrode surfaces are low efficiency of the electrocatalysts, large overpotential and low selectivity [1,2,3,4]. Meanwhile, if low overpotential is required for CO and formate production, high overpotential is needed for deep electroreduction to high-value-added C2 products (e.g., C_2_H_4_, C_2_H_6_) [5,6,7]. Obtaining C2 molecules is particularly interesting due to their higher energy density and potential to serve as precursors for various e-fuels and commodity e-chemicals [3,8]. The major cause of the poor selectivity of Cu electrodes for CO2ER to hydrocarbons could be the similar range of redox potentials for different reaction products, while the parasitic hydrogen evolution reaction (HER) contributes to the poor Faradaic efficiency (FE) of specific products [4,9]. The lower solubility of CO_2_ in aqueous solutions is an additional concern that limits the FE of CO2ER [1,10].

To achieve high efficiency in Cu-catalyzed CO2ER, the electrodes must have a sufficient number of active sites. Simple polycrystalline Cu electrodes have a rather small real surface area (*S_r_*), resulting in low efficiency [10,11]. Meanwhile, the selectivity and activity of Cu can be optimized by adjusting surface roughness, the size, shape, and interparticle distance of the Cu crystallites, as well by selecting the single crystal facets of the metal electrode [11,12,13,14]. An important way to increase the number of active sites of Cu-based catalysts towards CO2ER is to prepare a nanostructured electrode with high *S_r_* values. Since several studies have shown that Cu electrodes with high surface areas produce C2 compounds more efficiently; therefore, the influence of the roughness or specific nanostructures of the Cu electrode surface is of great importance [10,15,16,17,18].

C-C coupling is the most important step in the formation of C2 compounds; therefore, understanding the reaction pathway is critical for modulating C2 product selectivity and activity [19,20,21]. The activity of Cu porous structures as an electrocatalyst for CO2ER was found to depend on pore size. Meanwhile, the formation of C2 products predominates over C1 products when the width and depth of the pores on the Cu electrode are narrowed and increased [1,22]. An explanation was proposed that this phenomenon might be related to the extent of local alkalinization [23].

The dependency of electrocatalytic processes on the crystallographic orientation of an electrode surface is a well-established guiding principle in fundamental electrochemistry research and has been demonstrated for numerous materials and reactions [1,13,24,25]. Polycrystalline Cu contains many different crystal facets, and these facets strongly contribute to the CO_2_ reduction mechanism and product distribution [1,23,26,27,28,29]. Theoretical calculations suggest that the Cu(100) facet can significantly lower the dimerization energy barrier [27,30], while experimental studies have demonstrated that cube-like structures, such as the Cu(100) facet, promote ethylene production and the Cu(111) facet promotes methane production [18,31]. Therefore, since the dimerization reaction is facet-sensitive, another way to promote C2 formation is to adjust the facets of Cu-based catalysts. Thus, designing Cu catalysts with Cu(100) as the dominant exposed facet or reinforcing Cu(100) while weakening Cu(111) is an effective approach to improving selectivity toward C2 [1].

An effective way to increase the number of active sites of Cu-based catalysts towards CO2ER is to prepare a nanostructured Cu electrode [32,33]. A variety of methods are available to form nanostructures, and electrodeposition is one of them. Three-dimensional nano-ramified Cu three-dimensional electrodes or foams can be produced by metal electrodeposition accompanied by intensive hydrogen evolution [34,35,36]. This results in a large number of connected and unconnected pores that are evenly distributed within the Cu matrix [34,37]. The main advantage of this method is that it produces metal micro/nanostructures where the morphology and/or structure of deposits can be controlled by adjusting the electrodeposition conditions, such as current density, deposition temperature, pH, and electrolyte composition [35]. Cu 3D structures with high *S_r_* values are used in many applications, including CO_2_ reduction [38]. However, the potential of such Cu 3D electrodes in electrocatalytic practice, specifically in relation to the electrochemical transformation of CO_2_ to C2 products, has not been studied enough.

A key challenge for the practical application of CO2ER is the development of advanced catalysts [1]. This study is focused on strategies to improve the catalytic performance of Cu 3D base layers toward C2 selectivity in CO2ER. We deposited Cu foam electrodes with different structural parameters from acidic sulfate solutions that varied in the concentrations of the principal components, H_2_SO_4_ and CuSO_4_, as well as in the presence of Cl^−^ ions. Understanding the structure–activity relationship required obtaining a detailed and accurate image of the electrocatalyst, including its morphology, microstructure, and crystal facets, to ensure optimal CO_2_ reduction towards C2 products. Variations in the investigated electrode *S_r_* values allow us to determine the optimal roughness for optimizing C2 product efficiency.

## 2. Materials and Methods

### 2.1. Electrode Preparation

A 1 cm^2^ polycrystalline copper foil was used as the substrate for preparing the sample. First, a 8 µm thick compact Cu layer was deposited from an acidic sulfate solution [39]. Next, 3D Cu electrode structures were deposited from electrolytes with different compositions (Table 1). According to the results of our previous studies [40], all samples were deposited at a cathodic current density of 3 A·cm^−2^ for 20 s.

Analytical-grade chemicals H_2_SO_4_ (98.0%, pure p.a., Chempur), CuSO_4_ (≥99%, pure p. a., Chempur), HCl (36.4%, Reag. Ph. Eur., VWR Chemicals, Sanborn, NY, USA) and deionized water were used to prepare the electrolytes.

### 2.2. Electrochemical Measurements

Electrochemical measurements were carried out at ambient temperature in a three-electrode electrochemical cell using a Pt (2 × 2 cm^2^ strip) counter electrode, an Hg/HgSO_4_ reference electrode, and an AUTOLAB 302 potentiostat/galvanostat. The electrolytes were purged with Ar (99.990%, Elme Messer, Tallinn, Estonia) gas for at least 20 min prior to measurements. All potentials in the text are reported versus a standard hydrogen electrode (SHE).

The electrochemically active surface area (*ES_r_*) and surface roughness factor (*f_R_*) were assessed by double-layer capacitance measurements [39]. Cyclic voltammograms (CV) were recorded in a 0.1 M NaOH (≥97.0%, ACS reagent, Sigma-Aldrich, St. Louis, MO, USA) electrolyte at different scan rates in non-Faradaic regions. The double-layer capacitance values were determined by plotting the capacitive current values obtained at −0.50 V potential against the scan rate. The slope of the resulting linear relationship provided the double-layer capacitance value (*C*). *ES_r_* and *f_R_* values were calculated from the equations:

(1) $${ES}_{r}=\frac{C}{C_{sp}}$$

(2) $$f_{R}=\frac{{ES}_{r}}{S_{G}}$$

where *ES_r_* is the electrochemically active real surface area, cm^2^; *C* is the copper electrode double-layer capacitance, mF; *C_sp_* is the specific double-layer capacitance of copper in an alkaline solution equal to 0.02 mF cm^−2^ [41]; *f_R_* is the surface roughness factor; and *S_G_* is the geometrical surface area of an electrode, cm^2^.

Electrocatalytic activity of the Cu 3D electrodes in the CO2ER process was measured in a gas-tight H-cell. The anode was a Pt plate, the cathodes were different Cu electrodes with a nominal surface area of 0.63 cm^2^, and the electrode potential was measured with respect to Hg/HgSO_4_ reference electrode. The anodic and cathodic compartments were separated by an anion-exchange membrane (Nafion 117, Sigma-Aldrich St. Louis, MO, USA). Both compartments were filled with 65 mL of 0.1 M KHCO_3_ (≥99.7%, p. a., ACS, Carl Roth, Karlsruhe, Germany). Prior to the measurement, the electrolyte was purged with Ar or CO_2_ for 20 min. The CV curves were recorded by scanning the potential from −0.6 V to −2.0 V at a rate of 5 mV s^−1^. Before each measurement, the electrolyte was saturated with CO_2_ gas (99.998%, Elme Messer Tallinn, Estonia) for 20 min at a 30 mL·min^−1^ rate to reach pH = 7. All experiments were performed at 298 K and constant stirring and were performed at least in triplicate.

The determination of the different facet distribution of the investigated Cu 3D electrodes was based on Pb underpotential deposition (UPD) voltammetric measurements in the solution of 0.1 M KClO_4_ (99%, Thermo Scientific, Waltham, MA, USA) + 2 mM NaCl (≥99.5%, ACS, Merck, Darmstadt, Germany) + 2 mM PbCl_4_·3H_2_O (99%, Thermo Scientific)+ 1 mM HClO_4_ (60%, ACS reagent, Sigma-Aldrich St. Louis, MO, USA) [42,43,44]. In accordance with the previous studies [39], the Pb UPD deposition time for a monolayer formation was set to 900 s, which was followed by a cathodic potentiodynamic polarization between −0.11 V and +0.17 V at a scan rate of 5 mV s^−1^. In order to determine the distribution of facets of Cu 3D electrodes, the obtained polarization curves were fitted to a Gaussian mathematical function [44]. The peak potential values in the lead UPD CVs recorded on individual Cu facets were used to assess the facet distribution on copper surfaces deposited from different solutions [42].

Modification of the Cu facet distribution or re-faceting of a compact (non-porous) Cu electrode was performed in a 0.1 M NaCl electrolyte at a 500 mV·s^−1^ scan rate between Cu oxide reduction potential −0.76 V and selected oxidation potential 1.24 V [42]. The potential cycling was stopped at the reduction potential of −0.76 V. After modification of the facets, the electrode was consecutively cycled in the potential region prior to the oxidation of copper with a scan rate of 500 mV s^−1^ to remove traces of copper chloride or copper oxide passivating the surface.

### 2.3. Morphological Characterisation Using Scanning Electron Microscopy (SEM)

The Helios Nanolab 650 (FEI) two-beam system was used in secondary electron mode at a 3 kV acceleration to study the morphology of Cu 3D layers. The porosity, pore and dendrite size of the Cu 3D layers were assessed with a help of ImageJ 1.54d software.

### 2.4. CO_2_ Reduction Gaseous Product Analysis

The outlet of the cathodic electrochemical cell compartment was connected to a gas chromatograph (GC-2030, Shimadzu, Kyoto, Japan) for periodical sampling of the gaseous product of CO_2_ reduction. The GC was equipped with a dielectric-barrier discharge ionization detector (BID), and helium (≥99.9999%, Elme Messer) was the carrier gas. The GC was calibrated regularly using standard gas mixture (Elme Messer Tallinn, Estonia) under standard conditions (1 atm, 298 K). A typical CO_2_ electroreduction experiment at a constant applied voltage spanned over 120 min. A total of six gas aliquots (1 cm^3^ each) were measured. They were injected into the GC every 15 min. The first injection was performed 5 min after the start of the CO_2_ reduction reaction; this ensured adequate flushing of the transfer line of atmospheric contaminants. The GC data collected in this work were translated to Faradaic efficiencies (FE) according to the following equation:

(3) $$FE=\frac{e_{output}}{e_{input}}\times 100\%=\frac{y\times z}{\frac{Q}{F}}\times 100\%=\frac{y\times z\times F}{Q}\times 100\%$$

where *e_output_* is number of moles of electrons required for reducing CO_2_ to a particular product; *e_input_* is total number of moles of electrons measured during the sampling period; *Q* is measured charge, C; *F* is Faraday’s constant, 96,485 C·mol^−1^; *y* is experimentally determined amount of product, mol; and *z* is the number of electrons required to obtain 1 molecule of product [45].

Production of H_2_ during CO_2_ reduction was analyzed with an H_2_ sensor (Unisense, Aarhus, Denmark). Calibration of the sensor was performed using a standard H_2_ and Ar mixture. H_2_ concentration in the CO_2_ reduction gas mixture was monitored continuously throughout the measurement. We performed three to five FE measurements for each sample.

## 3. Results and Discussion

### 3.1. Cu Foam Microstructural Characterization

Three-dimensional (3D) crystalline Cu structures, or foams, can be produced by metal deposition in an acidic sulfate solution at current densities greater than 0.5 A·cm^−2^ [35,46]. Considering the importance of metal microstructure on CO2ER [1,12,13] and the need to evaluate how structural parameters influence catalyst activity in producing C2 products, Cu foam samples were deposited in solutions with varying concentrations of H_2_SO_4_ and CuSO_4_, with and without Cl^−^ ions present. Working electrodes (CuI–CuVIII) were deposited for further investigations, and the nomenclature of samples is presented in Table 1.

**Figure 1:**
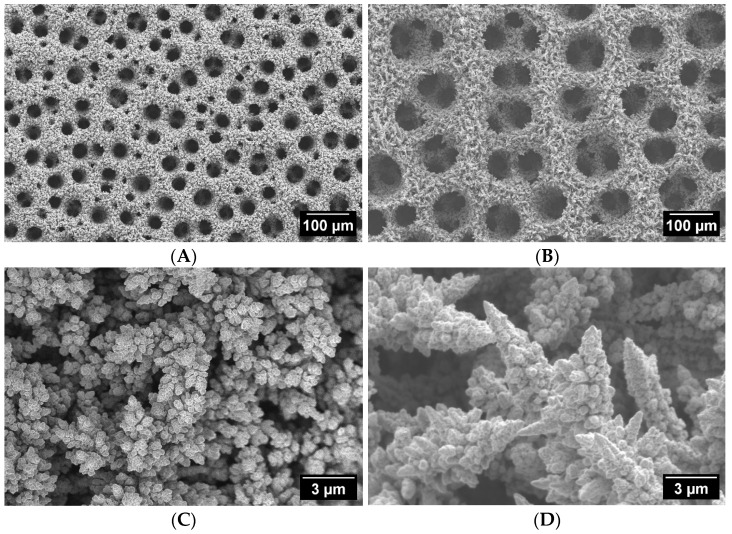
SEM images at different magnifications of Cu 3D structures deposited in solutions: 0.2 M CuSO_4_ + 1.5 M H_2_SO_4_ (**A**,**C** [40]) and 0.4 M CuSO_4_ + 1.5 M H_2_SO_4_ (**B**,**D**).

**Figure 2:**
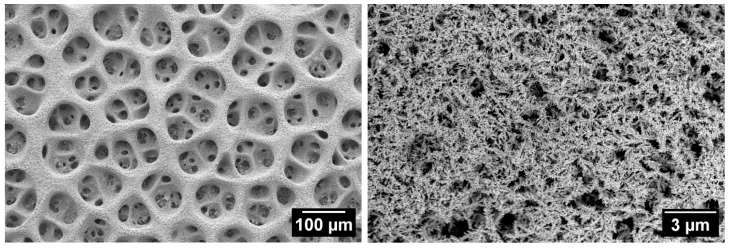
SEM images at different magnifications of Cu 3D structure deposited in solution of 0.2 M CuSO_4_ + 1.5 M H_2_SO_4_ + 50 mM HCl.

Figure 1 and Figure 2 show typical SEM images at different magnifications, demonstrating the surface morphology of Cu foam samples deposited in solutions with and without Cl^−^. The macro topography of Cu 3D structures deposited in the presence of Cl^−^ ions was very similar for all the samples that were investigated; therefore, only one sample from this group is presented in Figure 2. Meanwhile, Figure 1 shows images indicating apparent structural differences for samples deposited in solutions without Cl^−^ additives.

It has been proven that the formation of the foam structure is caused by the competitive reaction of Cu deposition and HER, resulting in the formation of 3D morphology with a unique pore size distribution and highly porous dendritic walls [34,35,37]. The hydrogen evolution reaction creates two types of pores [40]. The first type is macropores, or holes, formed by detached hydrogen bubbles. The second type originates from hydrogen bubbles generated at the tops of copper grain agglomerates during growth [41]. Meanwhile, the walls of the deposited 3D structures are composed of ramified dendrites that extend in all directions and cross-link with each other. This results in a loose structure with empty spaces (for example Figure 1D).

The number and size of the macropores, as well as the width of the walls between them, depend primarily on the presence of Cl^−^ ions rather than on the sulfate-to-sulfuric acid ratio of the depositing solution. Table 2 summarizes the results of the electrolyte composition’s effect on porosity parameters.

The macropores of the samples deposited in solutions without HCl were smaller and more uniform in size. Their average size ranged from 25 to 45 µm (Figure 1A,B). In contrast, adding HCl resulted in coalesced holes of various sizes (22–129 µm) with an average size of 65–80 µm (Figure 2). The CuI sample deposited in a solution without Cl^−^ ions had the lowest average macropore size (25.3 µm) and the highest average pore density (4.0 × 10^5^). Meanwhile, the other investigated samples had similar values for this parameter, ranging from 1 × 10^4^ to 2 × 10^4^.

As-deposited copper foam walls exhibit a dendritic morphology with numerous secondary and higher-order branches (Figure 1C,D and Figure 2). The average length and diameter of the primary branches depend on the concentrations of acid and sulfate, as well as the presence of Cl^−^ ions in the deposition solution. Cu foam walls formed in a solution without Cl^−^ ions consist of branches that range in length from 7 to 14 µm and in width from 3 to 4 µm. A more spatially dense structure with smaller branches was observed for the CuI sample (Figure 1C), which was deposited in a solution containing 1.5 M H_2_SO_4_ and 0.2 M CuSO_4_. In contrast, Cu foams with larger branches were obtained in solutions containing 0.4 M CuSO_4_ (e.g., sample CuII, Figure 1D).

Adding Cl^−^ ions to an acidic sulfate solution accelerates the metal deposition reaction. This results in more effective filling of the foam walls with Cu deposits compared to the absence of Cl^−^ ions. As can be seen in the SEM image in Figure 2, the additive dramatically reduces the size of the Cu crystallite aggregates forming the branches of the foam. The length and width of these branches are 1.2–2.4 µm and 200–300 nm, respectively. As the dendrite branches decreased in size, they became tightly packed, increasing the foam wall’s compactness. High-magnification images confirm the nanostructured nature of the pore walls, an essential factor in determining *S_r_* values of Cu foam sample.

The electrodeposited Cu 3D structures have a large surface area. A precise knowledge of this parameter is crucial for comparing the behavior of various catalytic systems. In applications involving electrochemical reactions, the electrochemically active surface area (*ES_r_*) is the key parameter because it is the area that transfers charge to species in solution [6]. *ES_r_* depends on how well the electrolyte accesses the pores and is influenced by surface roughness. According to the results of our previous studies dedicated to investigating the suitability of different *ES_r_* determination methods for porous 3D Cu structures, we applied double-layer capacitance measurements using cyclic voltammetry for this purpose. The obtained surface roughness factor values *f_R_* (the ratio of *ES_r_* to the geometric area of the sample, *S_G_*) are listed in Table 2.

The pore size distribution and compactness of the Cu crystallite agglomerates that form the walls between the holes determine the uniformity of these structures and the *ES_r_* values of the samples. For Cu foams deposited in solution without Cl^−^ additives, the *f_R_* values varied between 800 and 1000. Additionally, a higher concentration of CuSO_4_ in the deposition solutions (samples CuII and CuIV) increases the reduction of Cu ions relative to hydrogen evolution, wall thickness, and branch dimensions, as well as the *f_R_* values of these samples. In contrast, the *f_R_* values of samples deposited in solutions with Cl^−^ additives were two to three times higher than those of samples deposited in solutions without Cl^−^ ions. These values varied between 1600 and 2500. The specific surface area of Cu electrodes appears to be determined by the size of Cu grain agglomerates in the walls and the layer’s average thickness. The presence of Cl^−^ ions in the solution reduces the size of dendritic branches and, according to our previous studies [40], increases the average thickness of the Cu 3D layer from ~80 µm for samples deposited without Cl^−^ ions to ~120 µm.

### 3.2. Activity and Selectivity of Electrocatalytic CO_2_ Reduction on Cu Foam Electrodes

**Figure 3:**
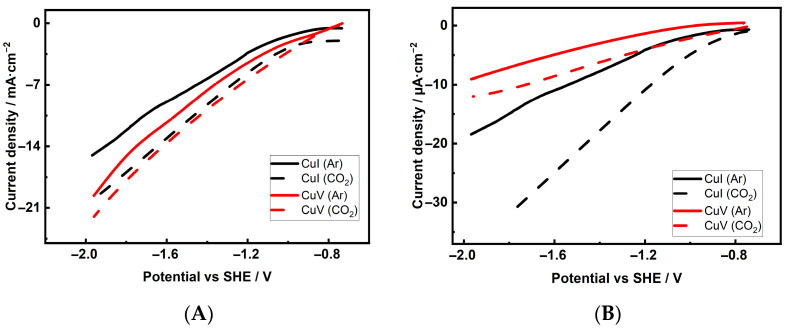
Linear sweep voltammetry curves of CuI (black curves) and CuV (blue red curves) electrodes in a 0.1 M KHCO_3_ solution saturated with Ar (solid curve) or CO_2_ (dashed curve). (**A**)—current density normalized by geometrical electrode surface; (**B**)—current density normalized by electrochemically active real surface area. Potential scan rate 5 mV s^−1^.

Linear sweep voltammetry tests were performed with the investigated Cu foam electrodes in a 0.1 M KHCO_3_ solution saturated with either Ar or CO_2_. The polarization curves were measured and are presented for one sample from each group in Figure 3A. Cathodic current densities increase progressively as the applied potential increases for both electrodes exposed to saturated solutions. However, within the negative potential range, it is clear that the Cu foam electrodes in the CO_2_-saturated medium exhibit higher cathodic current densities than in the Ar-saturated medium. Under an Ar atmosphere, the cathodic current is dominated by the H_2_ evolution reaction [1,21,23], while the increased current density is entirely due to the electrochemical reduction of CO_2_ in media saturated with this gas. Comparing the polarization curves of the first group (CuI–CuIV) and the second group (CuV–CuVIII) indicates that CO_2_ reduction is faster on Cu electrodes deposited in the presence of Cl^−^ ions. The higher observed reaction rates on these electrodes can be attributed to the increased *ES_r_* compared to samples deposited in a solution without additives (Table 2).

**Figure 4:**
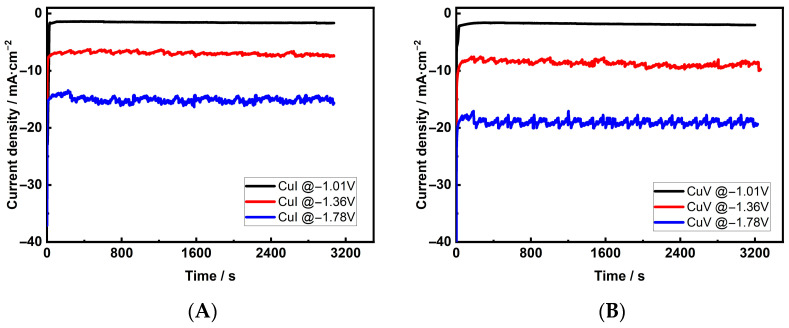
Current density over time curves of CuI (**A**) and CuV (**B**) electrodes at different potentials in a 0.1 M KHCO_3_ solution saturated with CO_2_.

Recent studies have shown that a Cu electrode converting CO_2_ to hydrocarbons and oxygenates requires a potential in the range of −0.8 V to −1.8 V [13,47]. Thus, the activity and selectivity of the investigated Cu foam samples as CO_2_ reduction catalysts were tested at fixed potential values of −1.01 V, −1.36 V, and −1.78 V, and the CO_2_ reduction process was carried out for 120 min under potentiostatic conditions. The chronoamperometric curves for the Cu 3D electrodes are presented in Figure 4. No significant changes were observed in the current densities for both electrodes, deposited in solutions without and with Cl^−^ ions, indicating the durability of the electrodes during the 2 h of cathodic performance. The fixed cathodic current densities of all investigated Cu electrodes are shown in Table 3. The major gaseous products of CO_2_ reduction (CH_4_, C_2_H_4_, and C_2_H_6_) were analyzed using online gas chromatography, and H_2_ was analyzed using a special sensor.

Among the Cu foam samples deposited in solutions containing Cl^−^ ions, the majority exhibited higher average geometric current densities (*j_g_*—current per geometrical area of electrode (0.63 cm^2^)) than samples deposited in solutions without the Cl^−^ additive. At a fixed potential of −1.36 V, the *j_g_* values of Cu electrodes deposited in a Cl^−^-free solution varied between 7.2 and 8.4 mA·cm^−2^. In contrast, the *j_g_* values of samples deposited in the presence of Cl^−^ ions ranged from 8.9 to 13.0 mA·cm^−2^. The higher reaction rates of CO_2_ reduction observed on the electrodes deposited in the presence of Cl^−^ ions can be attributed to the higher *ES_r_* values of the samples. However, when the *j_g_* values at the aforementioned potential are compared separately for Cu samples deposited from different types of electrolytes (with and without Cl^−^), the maximum *j_g_* values correspond to the CuI and CuVII samples, which do not have the highest *ES_r_* values in each group. Similar tendencies were observed for the other two applied CO_2_ reduction potentials.

To assess the overall electrode performance, it is useful to consider the normalized current density by *ES_r_* values of the electrode (*j_Sr_*). This allows one to compare the activity of catalysts with different roughness factors *f_R_* and determine whether higher catalytic activity results from a higher average turnover frequency or an increased number of active sites. The polarization curves for one sample from each group normalized to *ES_r_* are presented in Figure 3B.

Comparing the *j_Sr_* values of samples deposited from different electrolytes reveals that Cu foam electrodes deposited from the solutions without Cl^−^ additives are generally more active than those deposited in the presence of Cl^−^ ions. The *j_Sr_* values of the former varied from 11.0 to 16.3 µA·cm^−2^ at a fixed potential of −1.36 V, while the *j_Sr_* values of the latter varied from 5.6 to 11.6 µA·cm^−2^. Cu foam samples deposited in a solution containing 1.5 M H_2_SO_4_ and 0.2 M CuSO_4_ (sample CuI) or 0.8 M H_2_SO_4_ and 0.2 M CuSO_4_ (sample CuIII) exhibited the highest activity for CO_2_ reduction. Regarding the activity of Cu foam samples in CO2ER, the samples with the highest *f_R_* values were the least active in both groups, regardless of whether they were deposited with or without Cl^−^ ions. These results suggest that *ES_r_* is not the decisive factor in determining the CO_2_ cathodic reduction activity of Cu foam electrodes.

The selectivity of electrocatalytic processes is often described in terms of Faradaic efficiency, which is the fraction of the electrical current that goes toward producing a specific substance during steady-state electrolysis. The gaseous products of CO_2_ reduction were analyzed online, and the corresponding FE values were determined once the concentrations reached stable levels. The soluble products were not analyzed in this study.

The potential required for CO_2_ reduction is very close to that of the hydrogen evolution reaction. This means that the HER competes with CO_2_ reduction, negatively impacting energy efficiency and product selectivity [20,29]. Current efficiencies of H_2_ production on the investigated Cu electrodes were determined to range between 40% and 55% and were slightly higher at the more negative potential value of −1.78 V. Accordingly, no C2 or CH_4_ gaseous products were detected on any of the Cu foam electrodes tested at the initial potential of −1.01 V. Therefore, the detailed analysis of CO_2_ gaseous reduction products at this potential was not performed.

**Figure 5:**
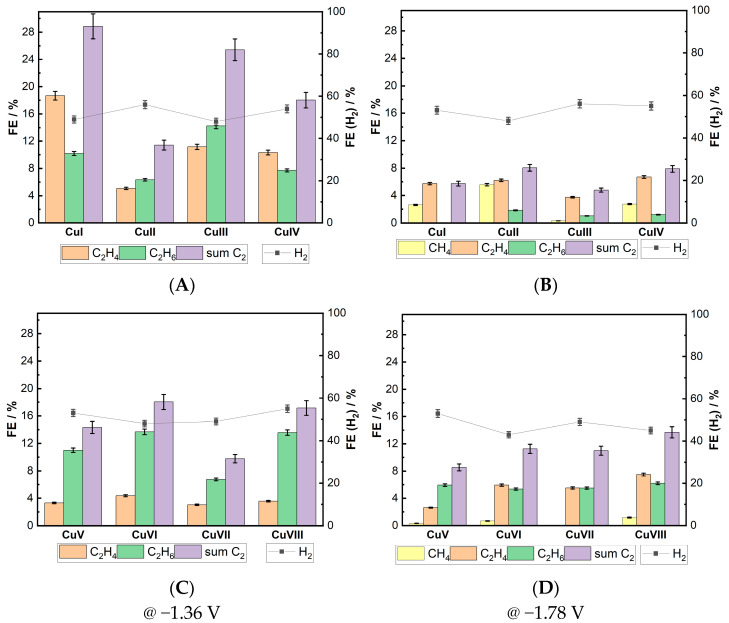
Faradaic efficiencies of gaseous products formed during 2 h of potentiostatic polarization of Cu electrodes at −1.36 V (**A**,**C**) and −1.78 V (**B**,**D**) in 0.1 M KHCO_3_ solution saturated with CO_2_.

Figure 5 illustrates the Faradaic efficiency distribution of gaseous products for various Cu foam electrodes at potential values of −1.36 V and −1.78 V. Methane was detected as a reaction product only at a polarization potential of −1.78 V. FE values for this compound were related to the electrolyte composition in which the sample was deposited. Cu electrodes deposited in a solution without additives appeared to be better catalysts for CH_4_ formation. The FE peak value was 5.6% for the CuII sample, whereas the FE value for the Cu electrodes deposited in the presence of Cl^−^ ions was 1.2% for the CuVIII sample. C2 products were generated on all of the investigated Cu foam electrodes under both −1.36 V and −1.78 V potentiostatic conditions. However, FE values were higher at −1.36 V than at −1.78 V. All of the electrodes subjected to cathodic polarization at −1.36 V generated a gaseous mixture of C2 hydrocarbons (C_2_H_4_ and C_2_H_6_), and the obtained results are further discussed in more detail.

A comparison of the activity and selectivity of the two types of Cu electrodes reveals that those deposited in a Cl^−^ free electrolyte are more active and have a structure that favors the formation of C2 gaseous compounds. FE values for C2 production in the first group of electrodes ranged from 12.5% to 28%. In contrast, analogous values for Cu structures deposited from an electrolyte containing a Cl^−^ additive varied from 10% to 18%. Comparing C2 gaseous product generation efficiencies in each group shows that in the first group, the CuI electrode at −1.36 V had the highest FE (28%) for C2 gaseous products, while the CuII electrode had the lowest (12.5%). In the second group, the CuVI and CuVIII electrodes produced C2 gaseous species most efficiently (FE ~17–18%), while the CuVII electrode was the least efficient (10%). FE values for CO_2_ reduction at a potential of −1.78 V did not exceed 12% for either group of samples. However, the decrease is more significant for the first group of electrodes.

A comparison of the ratios of the C2 gaseous compounds generated reveals that C_2_H_6_ dominates over C_2_H_4_ production at the second group’s electrodes at a potential of −1.36 V (Figure 5C). For the first group’s electrodes, however, this ratio is nearly equal, except for the CuI sample, which favors C_2_H_4_ formation (see Figure 5A).

Several authors have come to similar conclusions, indicating that increasing the surface roughness of the Cu electrode contributes to the C-C coupling reaction and results in a selective production of a C2 hydrocarbon mixture instead of methane [13,38]. However, our results show that the first group of Cu foam samples are more efficient at producing C2 gaseous compounds, despite having lower *f_R_* values than the second group of samples. Even when comparing FE values between the first group of electrodes, the electrode with the lowest *f_R_* value (CuI) has the highest FE of C2 gaseous compound production. This suggests that simply looking at the *ES_r_* values of Cu foam electrodes is not enough; the microstructural characteristics of the samples should be examined and their influence evaluated in more detail. This also implies that other structural factors, such as preferred faceting, grain boundaries, and spatial arrangement of Cu 3D structures, may significantly affect the CO_2_ reduction process.

### 3.3. The Structure–Properties Relationship

#### 3.3.1. Crystal Facet Effect

It is difficult, if not impossible, to unambiguously derive which structural elements on a polycrystalline catalyst surface, especially if it is a porous 3D structure, are contributing to the observed catalytic activity. This is because the measured catalytic activity is always a sum of activities resulting from different structures. Since morphology, microstructure, and crystal facets are the main structural parameters affecting the catalytic performance of investigated electrodes, we have attempted to assess the potential impact of each of them on CO_2_ reduction and C2 gaseous product yields.

X-ray diffraction (XRD) measurements are usually applied to evaluate the preferred crystal orientation of metal electrodes. However, the results for Cu foam samples are rather contradictory [37,48,49]. Conventional Bragg–Brentano geometry for XRD experiments has limited use for thin metal foam films because it is difficult to differentiate the metal foam’s contribution to peak intensity from that of the underlying metal substrate [46,50]. Meanwhile, several authors have demonstrated that Pb UPD can estimate the contribution of different facets or crystallographic domains in multifaceted Cu surfaces by decoupling peaks in cyclic voltammograms [42,43,44].

**Figure 6:**
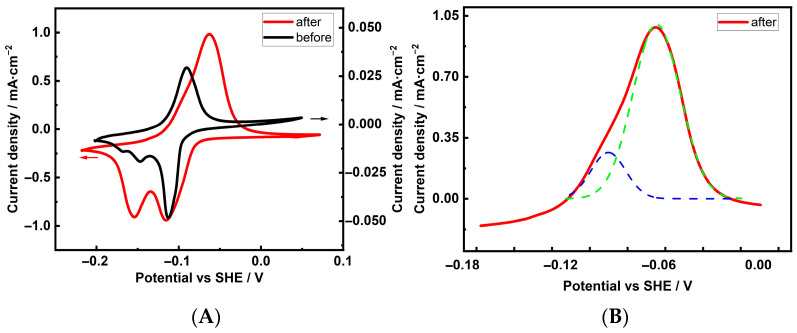
Cyclic voltammograms of Pb UPD deposition/dissolution on a base (BE) electrode before (black curve) and after re-faceting (red curves) in a 0.1 M NaCl solution (**A**) and (**B**) deconvoluted anodic dissolution curve of the re-faceted sample (blue curve corresponds to Cu(100) and/or Cu(110) facets, green curve corresponds to Cu(111) facet).

To establish a reference, the characterization of the Cu foam electrodes was started with the following Pb UPD experiment. Previous work established that an 8 µm Cu layer, electrodeposited from an acidic sulfate solution and serving as a base electrode (BE) for the formation of the Cu foam structure, exhibited a (100) preferred crystallite orientation [40]. Figure 6A (black curve) shows the Pb UPD cyclic voltammogram of the aforementioned electrode in a solution of 0.1 M KClO_4_ + 2 mM NaCl + 2 mM PbCl_4_·3H_2_O + 1 mM HClO_4_. In the anodic region, a sharp peak appears at −0.092 V. According to references [42,43], this potential is close to the Pb stripping potential on the Cu(100) facet. Electrochemical faceting of metals, based on applying different periodic potential routines, is an attractive procedure for developing textured metal surfaces [43,51]. Consecutive oxidation and reduction cycles at 500 mV·s^−1^ from −1.0 V to 2.0 V in a 0.1 M NaCl solution were performed with the aforementioned Cu BE electrode. The aim of this experiment was to reface the Cu electrode and evaluate the facet distribution using the Pb UPD technique. The most noticeable change in the striping of the re-faceted sample is that it displays an intensive, broad peak with a maximum at −0.045 V and a shoulder at −0.094 V (Figure 6A, red line). The presence of the latter indicates the existence of several single-facet peaks, which were decoupled using mathematical Gaussian functions. The obtained results are presented in Figure 6B. The smaller, convoluted peak at −0.094 V coincides with the peak corresponding to the initially present Cu(100) facets. Meanwhile, the broad peak at −0.075 V is close to the peak related to the Cu(111) facets, which appeared as a result of the re-faceting procedure.

**Figure 7:**
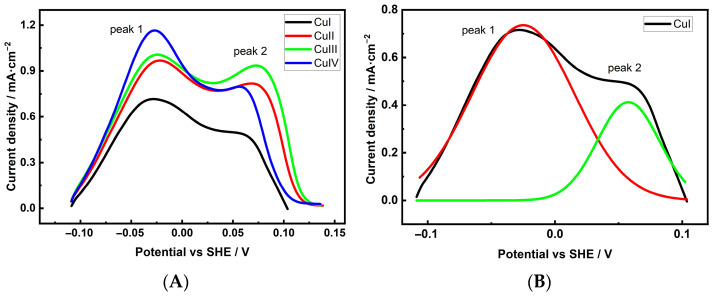
Stripping of the Pb UPD from Cu electrodes without Cl^−^ in the deposition solution (**A**) and deconvolution of the Pb UPD peak of the CuI electrode (**B**). Lines in the (**B**): red curve corresponds to Cu(100) and/or Cu(110) facets, green curve corresponds to Cu(111) facet.

**Figure 8:**
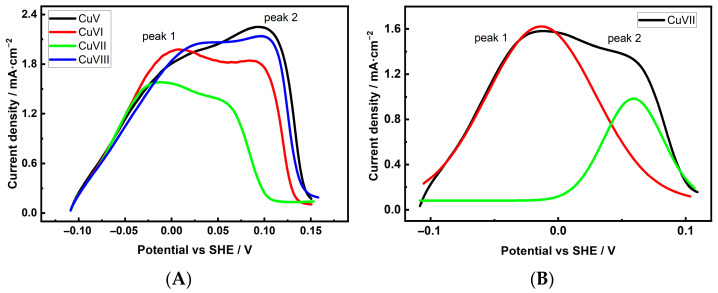
Stripping of Pb UPD from Cu electrodes with Cl^−^ in the deposition solution (**A**) and deconvolution of the Pb UPD peak of the CuVII electrode (**B**). Lines in the (**B**): red curve corresponds to Cu(100) and/or Cu(110) facets, green curve corresponds to Cu(111) facet.

Similar studies on Cu 3D electrodes note that the porous structure and large surface area of these samples require special consideration when performing Pb UPD measurements. Based on our experience applying these measurements to evaluate the *ES_r_* of Cu 3D structures [39], we determined that to maximize the surface coverage of Pb UPD layers, potentiostatic deposition of Pb UPD should be used for at least 900 s. Figure 7 and Figure 8, A and B, show the anodic dissolution (voltametric Pb stripping) curves of the Pb UPD layers formed on the investigated Cu foam electrodes, together with an example of deconvolution Figure 7 and Figure 8B.

Several patterns can be observed from the presented curves. First, unlike the Pb UPD dissolution curve of a non-porous electrode, the dissolution curves of Cu 3D electrodes shift to the positive potential range. The extent of the shift is related to the values of the sample *S_r_* and is more significant for samples with higher *S_r_* values. Next, the anodic dissolution curves of the Pb UPD layer of foam samples are clearly asymmetrical, consisting of at least two peaks that correspond to planes of two different orientations. According to the literature, Pb from Cu(111) facets dissolves last, i.e., at the most positive potentials, while Cu(100) and Cu(110) dissolve at more negative potentials [42,43]. Since XRD measurements detected the presence of Cu(111) facets in the Cu foam samples [40], it can be assumed that the second anodic peak in the more positive potential region of the stripping curves corresponds to Cu(111) facets. The first peak may correspond to (100) and/or (110), but separating them was not the focus of this study. While (111) facets, unlike (100) and (110) facets, are not favorable for C2 product formation during the CO2ER process, the main objective of this study was to evaluate the contribution of (111) facets to the overall facet composition and determine their potential impact on the reduction product composition. The integrated charge or fraction area of the facet peaks was estimated after deconvolution, and the obtained values are listed in Table 4.

When comparing the Cu(111) facet input for C2 product selectivity, it can be observed that the most effective electrode for C2 compound production, CuI, possesses the lowest number of (111) facets among the samples deposited in solution without an additive. Unfortunately, the differences in facet distribution among the samples are not significant enough to explain the variation in the activity and selectivity of Cu foam in the CO2ER process. Nevertheless, a clear correlation exists between the crystalline structure of the Cu foam electrodes and the CO_2_ reaction products with respect to CH_4_ production. The low proportion of Cu(111) facets may explain the low CH_4_ yields, particularly for samples deposited in the presence of Cl^−^ ions.

#### 3.3.2. Grain Boundaries and Defects

Defective site atoms are typically in an unsaturated coordination state; therefore, according to several studies, defects, including grain boundaries, play an important role in facilitating the C-C coupling in the CO2ER process [1,13,52,53]. Consequently, increasing the density of grain boundaries can significantly improve the activity and selectivity of the catalyst. From this perspective, comparing the fine structure of Cu 3D electrodes is appropriate.

In the field of XRD, the crystallite is considered to be the smallest single crystal or part of the grain that has no lattice defects and in which coherent scattering of X-rays takes place [54,55]. In certain cases, the grain may coincide with the crystallite. The grain size values of the Cu 3D structures were determined in our previous work based on XRD measurements, and it was found that the grain size values of the samples deposited in solutions without and with Cl^−^ ions are very close and vary in the range of 16.8–17.3 ± 5 nm [40].

**Figure 9:**
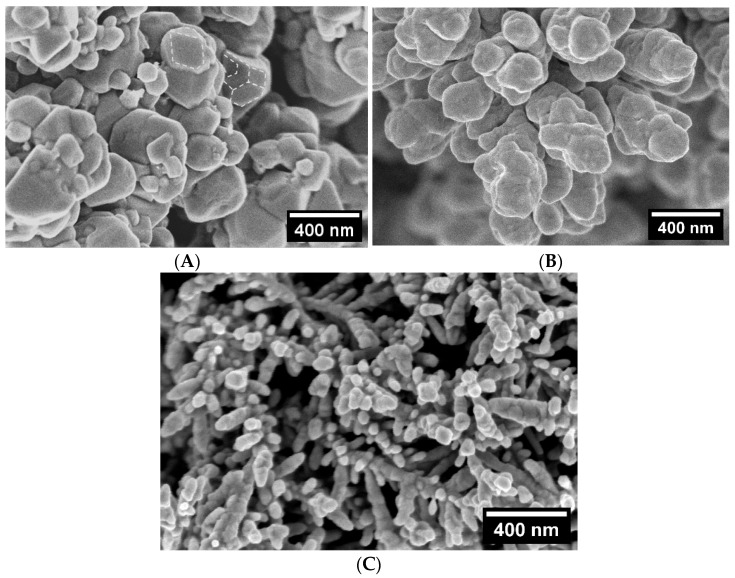
High-magnification (100,000×) SEM images of CuI (**A**), CuII (**B**), and CuV (**C**) electrodes.

High-magnification (100,000×) SEM images of Cu foam samples deposited in solution with and without chloride (Cl^−^) ions, presented in Figure 9, reveal that the 3D Cu material is composed of crystallites that “stick” together and can vary in size from a few tens to several hundred nanometers. Comparing the fine structure of the CuI and CuII samples, which exhibited different activity and selectivity for CO2ER, shows that the size of the large particles comprising both samples does not differ significantly, ranging from 250 to 300 nm. However, some CuI crystallites are covered with smaller particles up to 120 nm in diameter, which significantly increases the number of grain boundaries. Additionally, the crystallographic features of the CuI sample (Figure 9A) are clearly visible, indicating the edges of the crystallites and the planes they form. These features are highlighted for several crystallites in Figure 9A. Meanwhile, the CuII sample is composed of crystallites without clearly pronounced crystallographic features (Figure 9B). Despite the fact that the CuV sample is composed of branched, elongated crystallites with an average length of 72 nm and a diameter of 67 nm (Figure 9C), this structure is less effective in CO_2_ gaseous product yields than the CuI sample.

The fine structural characteristics observed in the investigated copper foam samples suggest that the CuI sample may have a higher density of structural defects than the other samples. This crystal structure could increase the number of active sites that promote CO_2_ ER. Additionally, catalysts with multiple edges and cores appear to be more effective at promoting C2 gaseous compound formation. This may explain why the CuI electrode exhibits higher efficiency in generating C2 gaseous products.

#### 3.3.3. Effect of Crystallite Dimensions and Geometry on C2 Yields

Several studies on CO2ER behavior have indicated that the process depends on the surface geometry of Cu catalysts [29,56,57] and that morphological features, such as pore size and shape, affect catalytic activity and selectivity [37,41]. However, our data suggest that macropore size and density have minimal impact on CO2ER yields. For instance, samples CuI and CuIII, which were deposited in an electrolyte without Cl^−^ ions, exhibit the highest FE values in this group of foams and possess reverse porosity parameters (Table 2).

Therefore, attention should be paid to the parameters of the crystallite aggregates forming the foam wall structure. When comparing the structural characteristics of the most and least efficient catalysts from the group of samples deposited in solution without the Cl^−^ additive, a few key factors should be considered. The CuI sample’s walls are composed of compactly packed crystallite aggregates that form branches with dimensions ranging from 4 to 7 µm in length and 2 to 3 µm in width. The gaps between the branches in this sample range from 0.5 to 1.0 µm, making the 3D structure narrow and dense (Figure 1C). In contrast, the CuII sample consists of larger aggregates ranging from 7 to 14 µm in length and 4 to 5 µm in width, with gaps between branches ranging from 1.5 to 2 µm (Figure 1D). Due to the larger aggregates forming the foam walls and the larger gaps between the branches, one might suppose that the CuII sample’s spatial structure is less compact than the CuI sample’s.

It is known that C-C coupling is the most important step in the formation of C2 compounds and that higher pH values in the reaction zone favor this reaction [23,25]. If structural features hinder the entry of HCO_3_^−^ into the reaction zone, the pH of the reaction medium remains elevated, which leads to C-C bonding and the formation of C2 products [23,38]. One might suppose that the denser spatial structure of the CuI electrode, compared to the CuII electrode, is more favorable for maintaining higher pH values in the pre-electrode layer and thus supporting C2 gaseous compound formation. This interpretation may explain why the FE value of C2 gaseous products for the CuI sample is as high as 28%, while for the CuII sample, it is only 12%.

The presence of Cl^−^ ions in the deposition solution of Cu foam structures reduces the size of dendritic branches and at the same time significantly increases *ES_r_* values with respect to samples deposited without the additive. The former conditions result in producing a dense spatial structure with the gaps between individual branches between 0.3 and 0.6 µm (Figure 2). However, these samples are not as effective as that of CuI for C2 gaseous compound production. The lower values of *j_Sr_* for foams deposited in the presence of Cl^−^ with respect to the CuI sample may indicate partial use of the potential active sites of the structure due to its microstructural features. In addition, caution should be exercised when comparing CO2ER performance between catalysts with different roughness factors because they will reach mass transport limitations at different rates. However, significant mass transport limitations can be reached for samples with extremely high spatial density and hence high *ES_r_* values when too little CO_2_ reaches the electrode surface. Mass transport modeling studies have largely explained this phenomenon, showing that an ideal compromise between pH increase and CO_2_ supply at the electrode surface gives optimal C2 selectivity on rough electrodes [13]. Therefore, one might suppose that the gap size between individual branches of foams deposited in the presence of Cl^−^ may be too small to be effective for production of C2 compounds.

The presented examples demonstrate the significant influence of the size of Cu foam branches, gaps between them, and the resulting spatial structure on its effectiveness in reducing CO_2_ to C2 gaseous compounds. A special configuration of branches typical for samples deposited in solution without Cl^−^ additive and containing a lower (0.2 M) concentration of CuSO_4_ forms a denser spatial structure that favors C2 gaseous product formation during CO2ER on Cu foam electrodes.

## 4. Conclusions

The structural diversity of Cu 3D samples electrodeposited from acidic sulfate solutions with varying concentrations of the principal components (H_2_SO_4_, CuSO_4_, and Cl^−^ ions) enabled the evaluation of the structure–property relationship of catalyst activity and selectivity toward C2 gaseous compound production during CO_2_ electrochemical reduction. Comparing the structural parameters of samples with the highest and lowest C2 gaseous product generation efficiency revealed that pore size and density have little influence on C2 gaseous product yield. The Cu foam sample that was deposited in a solution containing 1.5 M H_2_SO_4_ and 0.2 M CuSO_4_ was the most effective catalyst for producing C2 gaseous compounds. This is primarily due to the size of the nanostructured branches and the gaps between them. These features form a denser spatial structure than the other investigated electrodes, which were deposited in a solution without the Cl^−^ additive. This structural characteristic likely favors the maintenance of higher pH values in the pre-electrode layer, supporting C-C coupling and C2 gaseous compound formation. The higher catalytic activity and selectivity of this electrode could additionally be related to the lower contribution of the Cu(111) facet to the overall facet distribution and the higher density of structural defects.

The presence of Cl^−^ ions in the deposition solution of Cu foam structures decreases the size of the dendritic branches and the gaps between them. At the same time, it significantly increases the electrode surface area (*ESr*) values compared to samples deposited without Cl^−^ additive. The shape and dimensions of elongated crystallites, together with notably smaller gaps between individual branches, create a spatial structure. Due to rising mass transport limitations, this structure is characterized by lower activity and selectivity in C2 gaseous compound production for electrodes deposited in a solution containing Cl^−^ ions compared to samples produced without this additive.

## Figures and Tables

**Table 1:** Composition of the Cu 3D structure deposition electrolytes.

Cu 3D Electrode	Composition of the Deposition Electrolyte		
	H_2_SO_4_, M	CuSO_4_, M	HCl, M
CuI	1.5	0.2	-
CuII	0.8	0.2	
CuIII	1.5	0.4	
CuIV	0.8	0.4	
CuV	1.5	0.2	0.05
CuVI	0.8	0.2	
CuVII	1.5	0.4	
CuVIII	0.8	0.4	

**Table 2:** The porosity and surface roughness characteristics of Cu foam electrodes.

Electrode	Average Pore Size, µm	Average Pore Density, cm^−2^	$\mathrm{Surface}\;\mathrm{Roughness}\;\mathrm{Factor}\;(f_{R})$
CuI	25.3 ± 1.26	4.0 × 10^5^ ± 1.4 × 10^4^	814 ± 29
CuII	45.3 ± 1.81	1.0 × 10^4^ ± 4.2 × 10^3^	1035 ± 83
CuIII	40.0 ± 1.83	1.0 × 10^4^ ± 4.1 × 10^3^	817 ± 65
CuIV	45.0 ± 2.16	1.1 × 10^4^ ± 3.8 × 10^3^	911 ± 74
CuV	65.4 ± 2.88	1.7 × 10^4^ ± 4.0 × 10^3^	2512 ± 247
CuVI	73.0 ± 3.21	1.2 × 10^4^ ± 7.2 × 10^3^	1614 ± 154
CuVII	79.4 ± 3.73	1.7 × 10^4^ ± 4.9 × 10^3^	1786 ± 168
CuVIII	64.0 ± 2.98	1.6 × 10^4^ ± 4.2 × 10^3^	2444 ± 219

**Table 3:** Current densities normalized to the geometrical (*j_g_*) and electrochemically active surface area (*j_Sr_*) of Cu foam electrodes for CO2ER performed in 0.1 M KHCO_3_ solution saturated with CO_2_ under potentiostatic conditions.

E, V	*j_g_*/*j_Sr_*	CuI	CuII	CuIII	CuIV	CuV	CuVI	CuVII	CuVIII
−1.01	*j_g_* (S_g_), mA·cm^−2^	−2.16	−2.00	−2.05	−2.43	−3.29	−3.52	−4.87	−3.22
	*j_Sr_* (S_r_), µA·cm^−2^	−4.21	−3.14	−3.88	−4.23	−2.08	−3.46	−4.33	−2.09
−1.36	*j_g_* (S_g_), mA·cm^−2^	−8.38	−7.19	−7.51	−7.86	−8.86	−10.62	−13.02	−10.51
	*j_Sr_* (S_r_), µA·cm^−2^	−16.34	−11.03	−14.58	−13.69	−5.60	−10.44	−11.57	−6.82
−1.78	*j_g_* (S_g_), mA·cm^−2^	−16.57	−15.27	−15.29	−15.81	−17.10	−20.84	−24.71	−18.11
	*j_Sr_* (S_r_), µA·cm^−2^	−32.30	−23.45	−29.65	−27.54	−10.81	−20.49	−21.96	−11.76

**Table 4:** Ratio between the deconvoluted peak integrated areas of Pb UPD dissolution.

Electrode	Integrated Peak 1 Area	Integrated Peak 2 Area	Peak1: Peak2
	@ −0.02 V	@ 0.06 V	
CuI	78.55 ± 3.14	21.35 ± 0.90	3.7: 1
CuII	71.84 ± 3.23	28.16 ± 1.30	2.6: 1
CuIII	69.00 ± 2.70	31.00 ± 1.21	2.2: 1
CuIV	72.45 ± 3.04	27.55 ± 1.16	2.6: 1
	@ 0.1V	@ 0.02 V	
CuV	82.17 ± 3.78	17.83 ± 0.82	4.6: 1
CuVI	82.78 ± 3.48	17.22 ± 0.73	4.8: 1
CuVII	75.31 ± 3.31	24.69 ± 1.09	3.0: 1
CuVIII	86.00 ± 3.87	14.00 ± 0.63	6.1: 1

## Data Availability

The original contributions presented in this study are included in the article. Further inquiries can be directed to the corresponding author.

## Abbreviations

The following abbreviations are used in this manuscript:

BE	Base Electrode
CO2ER	Carbon Dioxide Electrochemical Reduction
CV	Cyclic Voltammetry
FE	Faradaic Efficiency
GC	Gas Chromatography
HER	Hydrogen Evolution Reaction
SEM	Scanning Electron Microscopy
SHE	Standard Hydrogen Electrode
UPD	Underpotential Deposition
XRD	X-ray Diffraction
